# Are old running shoes detrimental to your feet? A pedobarographic study

**DOI:** 10.1186/1756-0500-4-307

**Published:** 2011-08-24

**Authors:** Ulfin Rethnam, Nilesh Makwana

**Affiliations:** 1 Department of Orthopaedics, Glan Clwyd Hospital, Rhyl, UK; 2 Department of Orthopaedics, Wrexham Maelor Hospital, Wrexham, UK

## Abstract

**Background:**

Footwear characteristics have been implicated in fatigue and foot pain. The recommended time for changing running shoes is every 500 miles. The aim of our study was to assess and compare plantar peak pressures and pressure time integrals in new and old running shoes.

**Findings:**

This was a prospective study involving 11 healthy female volunteers with no previous foot and ankle problems. New running shoes were provided to the participants. Plantar pressures were measured using the Novel Pedar system while walking with new and participants' personal old running shoes. Plantar pressures were measured in nine areas of the feet. Demographic data, age of old running shoes, Body Mass Index (BMI), peak pressures and pressure-time integral were acquired. The right and left feet were selected at random and assessed separately. Statistical analysis was done using the paired t test to compare measurements between old and new running shoes.

The mean peak pressures were higher in new running shoes (330.5 ± 79.6 kiloPascals kPa) when compared to used old running shoes (304 ± 58.1 kPa) (p = 0.01). The pressure-time integral was significantly higher in the new running shoes (110 ± 28.3 kPa s) compared to used old running shoes (100.7 ± 24.0 kPa s) (p = 0.01).

**Conclusion:**

Plantar pressure measurements in general were higher in new running shoes. This could be due to the lack of flexibility in new running shoes. The risk of injury to the foot and ankle would appear to be higher if running shoes are changed frequently. We recommend breaking into new running shoes slowly using them for mild physical activity.

## Background

Footwear characteristics have been implicated as a cause of foot pain [1]. Use of customised insoles was found to alleviate post work foot discomfort in healthy individuals whose jobs required long periods of standing and walking [2]. Ill fitting footwear has been associated with foot pain [3]. Individually fitted sport shoes were found to be effective in reducing the incidence of foot fatigue [4]. There is an association between using inappropriate footwear and injuries [5]. An association between injuries and the age of sport shoes has been reported [6]. The recommendations are that running shoes need to be changed every 500 - 700 kilometres as they lose their shock-absorbing capabilities [7]. Elevated plantar pressures cause increased foot pain in people with cavus feet [8].

Walking plantar pressures in running shoes need to be investigated. There are no pedobarographic studies in the literature that compare new with old running shoes. We hypothesized that old running shoes transmitted higher plantar pressures as compared to new running shoes. If so, are old running shoes detrimental to our feet? The purpose of this study was to see whether the mean peak pressures & pressure-time integrals exerted at the plantar surface of feet were higher when using old running shoes as compared to new running shoes.

## Findings

This experimental study was conducted at the Foot and Ankle Unit after obtaining approval from the research panel and the ethical committee (North East Wales ethical committee). 11 healthy female volunteers with no previous foot and ankle problems, assessed using the validated AOFAS (American Orthopaedic Foot and Ankle Society) clinical rating system, were recruited for the study. The subjects were selected from a single working environment so that the activity level was similar during most part of the day. They were provided with an information leaflet prior to recruitment and once recruited, informed consent was taken.

The old running shoes were personal shoes of the participants that varied among the study group. These had leather uppers and quarters with rubber soles. The insoles were worn out to a varying degree based on the age of the shoe. The participants were provided with new running shoes that were of the same brand and make to reduce variability. Standard commercially available sport shoes (Nike) measured to the foot size of the subjects was used. These shoes were classed as running shoes and had regular insoles. These running shoes had leather uppers and quarters with rubber soles with a lightweight midsole single density with no additional cushioning materials.

Subjects were given 2-4 weeks to break into the new running shoes and were advised to use them during most part of the day. This was done was to prevent friction burns (blisters) from the new shoes causing error in pressure measurement. The participants were assessed only after they could walk comfortably in the new shoes.

The study area was a 15 m straight pathway without any obstructions to enable uninterrupted walking. Plantar pressures were measured using the Novel Pedar system (Novel gmbh, Munich, Germany), which uses a matrix of multiple capacitance transducers, embedded into an insole as rows and columns. The insoles assess the distribution of pressures acting over the entire plantar surface of the foot simultaneously.

For the study the subjects had the data storage inbox secured to their waists connected to appropriate sized insoles inside their running shoes. The Novel Pedar insoles were inserted into the subjects' shoes above the insoles present in the shoes. They were made to walk at a steady pace on the path using the new and their own old sport shoes. Prior to obtaining the measurements, the subjects were allowed a period of 5 minutes where they could practise walking at a self selected speed to help them become familiar with the test procedure. The subjects were instructed not to look at the ground while walking. 5 trials each were recorded from both the new and old running shoes. The average of five readings for each participant during steady state walking was taken and 11 sets of data were analyzed. The following data was collected: Body Mass Index, age of old running shoes and plantar pressures. Pressure data from each trial were processed using the Novel-win software package. This software measures parameters using standardised masks that account for specific regions in the foot. Each foot was divided into 9 regions (masks). These were: Medial hind foot, lateral hind foot, medial mid foot, lateral mid foot, medial metatarsal head, mid metatarsal head, lateral metatarsal head, great toe and lesser toes.

For each foot region, the analyzed parameters were: peak pressures (kPa) and pressure-time integral (kPa s), i.e. the sum of peak pressure in each frame of foot contact multiplied by the time between scans. These parameters were measured in different regions of the foot using the masks and any difference in measurements were assessed (medial versus lateral side).

Statistical analysis was done using the paired t test to compare peak pressures & pressure-time integrals between the new & old running shoes. To examine the relationship between peak pressures and body mass index, pressure-time integral and body mass index, the Pearson's correlation coefficient was calculated. Differences between the new and old running shoes were considered significant if p < 0.05.

11 female subjects with mean age of 28 years (Range: 18-48) were included in the study. The mean body mass index (BMI) of the study group was 25.9 (Range: 20-35). The mean age of old running shoes used for the study was 120.5 weeks (Range: 78 - 156). (Table 1)

**Table 1:** Subject profile

Subject	Age	Height (cm)	Weight (Kg)	BMI	Age of old shoe (Weeks)	Age of new shoe (Weeks)
TR01	28	165	66	24	104	2

TR02	29	150	45	20	104	3

TR03	19	150	55	24	78	3

TR04	30	150	57	25	156	4

TR05	27	152.5	81	35	130	3

TR06	18	152.5	76	33	104	4

TR07	23	160	54	21	156	3

TR08	48	160	53	20	104	4

TR09	22	163	77	29	156	3

TR10	34	165	83	30	104	3

TR11	30	165.5	65	24	130	4

Peak pressures were higher in new running shoes beneath most regions of the foot except the forefoot region where old running shoes transmitted higher plantar peak pressures. These differences were statistically significant under the whole foot & the lesser toes respectively. (Table 2) Assessment of peak plantar pressures did not reveal any significant difference under the whole medial or lateral aspect of the foot. (Table 2)

**Table 2:** Peak pressures - Comparison between new & old running shoes

Foot region	New running shoes (n = 11)	Old running shoes (n = 11)	Paired t test p value
		
	Peak pressure (kPa)	Peak pressure (kPa)	
**Total object**	330.5 ± 79.6	304 ± 58.1	0.01

**Medial Hind foot**	245.6 ± 53.3	244.5 ± 45.1	0.45

**Lateral Hind foot**	245.4 ± 53.6	234.0 ± 35.1	0.07

**Medial mid foot**	145.1 ± 38.9	139.5 ± 35.2	0.25

**Lateral mid foot**	149.8 ± 43.6	135.4 ± 25.6	0.07

**Medial metatarsal**	235.9 ± 72.9	249.1 ± 89.9	0.07

**Mid metatarsal**	232.8 ± 51.9	238.6 ± 62.9	0.24

**Lateral metatarsal**	203.5 ± 51.3	207.5 ± 51.3	0.29

**Great toe**	275.3 ± 87.2	268.3 ± 62.1	0.29

**Lesser toes**	182.4 ± 63.9	196.1 ± 63.9	0.03

			

**Hindfoot**	245.5 ± 52.8	239.3 ± 40.2	0.13

**Midfoot**	147.4 ± 40.9	137.4 ± 30.4	0.06

**Forefoot**	225.5 ± 70.9	231.9 ± 72.2	0.08

			

**Medial**	225.5 ± 81.5	225.3 ± 79.3	0.49

**Lateral**	195.3 ± 59.5	193.3 ± 58.9	0.32

Pressure time integrals were significantly higher beneath most regions of the foot in the new running shoes except the lesser toes where these were higher in the old running shoes. (Table 3) Pressure time integral was significantly higher in the new running shoes under both the medial & lateral aspects of the whole foot. (Table 3) There was a positive correlation between the body mass index (BMI) and whole foot peak pressure & pressure time integral (r: 0.33 & 0.69 respectively).

**Table 3:** Pressure time integral - Comparison between new & old running shoes

Foot region	New running shoes (n = 11)	Old running shoes (n = 11)	Paired t test p value
		
	Pressure time integral (kPa s)	Pressure time integral (kPa s)	
**Total object**	110 ± 28.3	100.7 ± 24.0	0.01

**Medial Hind foot**	51.6 ± 11.2	49.2 ± 13.3	0.17

**Lateral Hind foot**	53.7 ± 12.6	48.6 ± 13.3	0.02

**Medial mid foot**	46.3 ± 15.1	40.8 ± 13.2	0.03

**Lateral mid foot**	53.3 ± 18.3	44.8 ± 11.7	0.01

**Medial metatarsal**	64.3 ± 27.5	55.3 ± 24.8	0.01

**Mid metatarsal**	66.2 ± 21.7	56.4 ± 17.9	0.01

**Lateral metatarsal**	65.2 ± 22.3	51.5 ± 15.8	0.004

**Great toe**	53.3 ± 18.2	55.0 ± 20.8	0.29

**Lesser toes**	41.3 ± 17.2	46.9 ± 23.3	0.04

			

**Hindfoot**	52.7 ± 12.1	48.9 ± 11.7	0.01

**Midfoot**	49.8 ± 12.4	42.8 ± 16.8	0.001

**Forefoot**	58.1 ± 20.3	53.0 ± 23.0	0.004

			

**Medial**	53.9 ± 19.0	50.1 ± 19.5	0.01

**Lateral**	53.4 ± 15.9	47.9 ± 19.3	0.003

Footwear has been implicated in causation of foot pain and injuries [1,3]. In a study on healthy individuals, customised insoles were shown to reduce post work discomfort [2]. Impaction on the ground would cause wear of the footwear resulting in a reduced shock-absorbing capability [9]. Individually fitted sport shoes with good shock absorbing capabilities were found to reduce foot pain and fatigue as compared to subjects own shoes [4]. For this reason regular replacing of shoes has been advised. Shoe age has been postulated as a cause for injuries [6]. The theory is that the cushioning properties of shoes reduce with increasing mileage [10]. Studies show that incorrectly fitting footwear is strongly associated with forefoot pathology and foot pain [3,11]. Other studies have shown the effects of footwear on feet especially in women [12,13].

There has been no study in the literature that compares the plantar pressures under the foot in new and worn-out trainers. The only study comparing new and worn shoes with regards to cushioning concluded that worn shoes caused increased stance phase and kinematic adaptations by the runner when shoe cushioning reduces [10]. The consensus is that worn-out running shoes offer less protection, as mentioned earlier, causing high plantar pressures [10]. This may directly or indirectly cause musculoskeletal problems. The Novel Pedar system has been found to be accurate in assessment of vertical force measurement [14,15].

Our subjects were made to walk at a steady relaxed pace to avoid higher plantar pressures during running or speed walking [16]. To reduce variability we chose our subjects from the same gender and provided them with same make of new trainers. Our study showed that peak pressures and pressure time integrals varied significantly between new and old running shoes. To our surprise the new running shoes seemed to transmit higher plantar peak pressures and pressure time integrals as compared to the old running shoes. The peak plantar pressures were significantly increased in new running shoes in the whole foot and the lesser toes. In the forefoot region the plantar pressures were more in the old running shoes, but this was not statistically significant. The pressure time integral was significantly increased in the new running shoes in most of the areas of the foot.

We speculate the reason for our findings to be the inherent stiffness of new shoes. When the leather in new shoes is not pliable, the feet may be held more firmly in the shoes. This may be correlated by our finding of increased contact times in new running shoes. In stiff & non-pliable shoes with increased contact times, the plantar pressures would be high. A "wearing in" period for new running shoes till the initial stiffness is overcome may prevent this increase in plantar pressures. In spite of having had a "run in" period, our subjects showed higher plantar pressures with new running shoes. This could have been a result of non-compliance of subjects to wear the new trainers for most part of the day as instructed or inadequacy in the length of the "wearing in period". A "wearing in" period of more than 4 weeks may be needed to reduce the stiffness of new running shoes. We feel that a "wearing in" period for running shoes would make them more flexible and therefore reduce contact times and subsequently the plantar pressures. The positive correlation between body mass index and peak pressures found in our study is well documented in the literature [17].

Limitations of our study was that we did not compare "like for like" running shoes and the sample size was small. Other limitations include sample convenience and using single gender subjects for the study. In light of the fact that there has been no similar study in the literature, this study has broadened our perspective and identified that more research is needed into this important issue. We recommend a "wearing in" period for new running shoes, alternating its use with used running shoes till the new running shoes are more flexible.

## Conclusion

Increasing musculoskeletal problems have been postulated due to use of inappropriate worn-out running shoes. This may not be completely true as our study revealed. Keeping a log of the mileage of running shoes, avoiding frequent change of running shoes and an adequate "wearing in" period for new running shoes would protect the foot from injuries related to inappropriate footwear. More research is needed into the effect of age of running shoes on plantar pressures as recommendations could help in prevention of foot problems related to inappropriate footwear during sporting activities.

## Competing interests

The authors declare that they have no competing interests.

## Authors' contributions

UR the main author for the manuscript and was involved in conducting the study, data acquisition and preparation of the manuscript. NM the senior author was involved in conception and design of the study. All authors have read and approved the final manuscript.
